# PCirc: random forest-based plant circRNA identification software

**DOI:** 10.1186/s12859-020-03944-1

**Published:** 2021-01-06

**Authors:** Shuwei Yin, Xiao Tian, Jingjing Zhang, Peisen Sun, Guanglin Li

**Affiliations:** National Engineering Laboratory for Resource Development of Endangered Crude Drugs in Northwest China, The Key Laboratory of Medicinal Resources and Natural Pharmaceutical Chemistry, The Ministry of Education, College of Life Sciences, Shaanxi Normal University, Xi’an, 710119 Shaanxi People’s Republic of China

**Keywords:** CircRNA, Machine learning, Plant, Random forest

## Abstract

**Background:**

Circular RNA (circRNA) is a novel type of RNA with a closed-loop structure. Increasing numbers of circRNAs are being identified in plants and animals, and recent studies have shown that circRNAs play an important role in gene regulation. Therefore, identifying circRNAs from increasing amounts of RNA-seq data is very important. However, traditional circRNA recognition methods have limitations. In recent years, emerging machine learning techniques have provided a good approach for the identification of circRNAs in animals. However, using these features to identify plant circRNAs is infeasible because the characteristics of plant circRNA sequences are different from those of animal circRNAs. For example, plants are extremely rich in splicing signals and transposable elements, and their sequence conservation in rice, for example is far less than that in mammals. To solve these problems and better identify circRNAs in plants, it is urgent to develop circRNA recognition software using machine learning based on the characteristics of plant circRNAs.

**Results:**

In this study, we built a software program named PCirc using a machine learning method to predict plant circRNAs from RNA-seq data. First, we extracted different features, including open reading frames, numbers of k-mers, and splicing junction sequence coding, from rice circRNA and lncRNA data. Second, we trained a machine learning model by the random forest algorithm with tenfold cross-validation in the training set. Third, we evaluated our classification according to accuracy, precision, and F1 score, and all scores on the model test data were above 0.99. Fourth, we tested our model by other plant tests, and obtained good results, with accuracy scores above 0.8. Finally, we packaged the machine learning model built and the programming script used into a locally run circular RNA prediction software, Pcirc (https://github.com/Lilab-SNNU/Pcirc).

**Conclusion:**

Based on rice circRNA and lncRNA data, a machine learning model for plant circRNA recognition was constructed in this study using random forest algorithm, and the model can also be applied to plant circRNA recognition such as *Arabidopsis thaliana* and maize. At the same time, after the completion of model construction, the machine learning model constructed and the programming scripts used in this study are packaged into a localized circRNA prediction software Pcirc, which is convenient for plant circRNA researchers to use.

## Background

Circular RNA (circRNA) is a newly identified kind of noncoding RNA. In contrast to typical linear RNA, it has no 5′ terminal cap structure or 3′ terminal poly-A tail structure but instead a closed-loop structure formed by the end-to-end connection of the 5′ terminus and 3′ terminus [1]. CircRNA was first found in plant viroids in the 1970s, but it was considered a by-product of transcription due to its low level of expression [1, 2]. In recent years, with the development of high-throughput sequencing and bioinformatics technology, a large number of circRNAs have been found from prokaryotes to eukaryotes, and some of them have been proven to encode proteins [3, 4].

Although there are many studies on circRNAs to date, most of them are concentrated in mammals and humans, and there are few studies on circRNAs in plants, such as *Arabidopsis thaliana*, *Oryza sativa*, *Triticum aestivum*, and *Solanum lycopersicum* [5–8]. Similar to animals, plant circRNAs can act as miRNAs and RNA binding protein (RBP) sponges. For example, circRNAs in grapes can be used as miRNA sponges [9, 10]. In addition, circRNAs can also respond to biotic and abiotic stresses on plants [9, 11]. For example, 163 circRNAs were differentially expressed in tomato under low-temperature stress [6].

The recognition of circRNAs is the basis of studying the function and regulation of circRNAs. Currently, CIRI [12], CIRCexplorer2 [13], and find_circ [14] are popular software programs for the identification of circRNAs. One of the important common foundation for the ability of these three software programs to predict circRNAs from transcriptome data is the supporting number of reads covering circRNA back-splicing junctions (BSJs). However, the empirical standard used for supporting the number of reads is different in each prediction software, which leads to a great difference in the number of predicted circRNAs, and only a small number of overlapping circRNAs are obtained by different software programs [15]. At the same time, because the expression of circRNAs varies in different stages and tissues, it is easy to lose some circRNAs by using the number of junction reads as a vital standard to predict circRNAs. To overcome the above shortcomings, a new animal circRNA identification method, DeepCirCode, which is based on a machine learning method, was developed and achieved good results in mammals [16]. The characteristics used by DeepCirCode include GT-AG splicing sites, Alu repeat sequences upstream and downstream of the back-splicing site, and sequence directions at both sites of the circRNA splicing junction that are opposite to those on the genome. However, using these features to identify plant circRNAs is infeasible because the characteristics of plant circRNA sequences are different from those of animal circRNAs; for example, plants are extremely rich in splicing signals and transposable elements, and their sequence conservation in rice, for example is far less than that in mammals [17]. To solve these problems and better identify circRNAs in plants, it is urgent to develop circRNA recognition software using machine learning based on the characteristics of plant circRNAs.

In this study, we first took circRNAs and lncRNAs as positive and negative sets, respectively, and built a machine learning model based on the main characteristics of k-mers, ORFs, and coding information of sequences covering back-splicing sites. Then, a tool named PCirc, which can be used in the prediction of plant circRNA, was developed and has achieved good prediction performance. Pcirc source code and installation instructions are available at https://github.com/Lilab-SNNU/Pcirc.

## Implementation

### Dataset

In this study, rice circRNA data were downloaded from PlantCircBase [18] (http://ibi.zju.edu.cn/plantcircbase/), and lncRNA data were downloaded from GreeNC (http://green.sciencedesigners.com/) [19]. To make the data set more credible, we first compared the circRNAs data with the lncRNA data, sifted out the sequences with sequence similarity higher than 95% in the two data sets, and then compared the sequences within each data set and removed those with similarity higher than 95%, keeping the longest one. Finally, we used the Box-whisker Plot method to remove the extreme data values (length too long or too short) from both data sets. In summary, 33,101 circRNAs and 4656 lncRNAs were obtained as positive and negative data, respectively. We randomly selected 4000 sequences were from the positive and negative data, then got a total of 8000 sequences as the training set, and the remaining data were used as an independent test set. The circRNAs and lncRNAs of *Arabidopsis* and maize were also downloaded from PlantCircBase and GreeNC, respectively, and then used to test the universality of Pcirc.

### Random forest

Machine learning (ML) is a multidomain interdisciplinary subject. It is the fundamental way to make computers intelligent, and its application is widespread throughout the fields of artificial intelligence and in the fields of biology and medicine [20–22]. In general, it is difficult for humans to obtain the required information directly from the original data. There are many ML algorithms in ML, such as random forest (RF), k-nearest neighbors (KNN), Support Vector Machine (SVM) and Gaussian naive Bayes (GNB).

Random forest (RF) is an aggregation of multiple unpruned decision trees from separate bootstrap samples of the training data and every feature subset sampled independently from the original feature space [23]. It can construct multiple independent decision trees from the original features of the training data set and then fuse all trees by voting to obtain an optimal classification model, which has been widely used in data processing fields, including bioinformatics [24, 25]. K-nearest neighbors (KNN) is another popular algorithm in the field of ML; although KNN can also handle classification problems well, many parameters in the algorithm need to be adjusted [26]. With increasing numbers of key parameters in KNN, the amount of calculation will expand rapidly, while RF requires the adjustment of only a few model parameters to obtain a good prediction classification model [27].

### Experimental setting in Pcirc

**Fig. 1:**
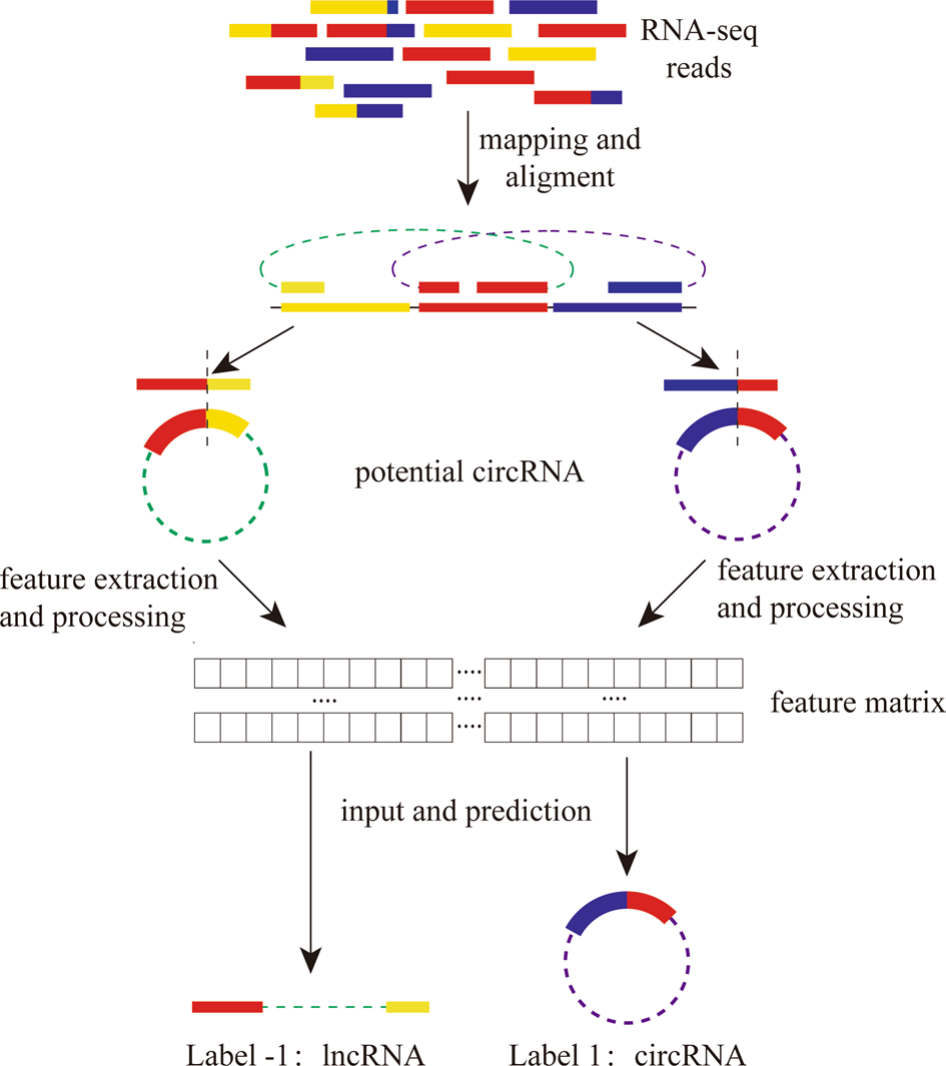
The process of PCirc

In this study, we first built the ML model by using a Python module named scikit-learn (https://scikit-learn.org/) [28], an ML module that includes many ML algorithms. Then, we developed the software Pcirc, which is based on the pipeline shown in Fig. 1.

In scikit-learn, we set the parameters for the ML algorithms as follows. For RF, the number of trees in the forest (n_estimators) was set to 100, and the other parameters were kept at the default values. GNB, the default parameters were used. For SVM, the parameter hash was {C:10, gammer:0.001, kernel: ‘rbf’}.For KNN, the parameter hash was {n_neighbors:7, weights: ‘uniform’, p:2} and the other parameters were set to the default values.

### Feature extract

Extracting features with recognition ability from the training set is a key step in building an ML model. Some simple single features, such as GC content and sequence length, cannot easily and directly distinguish between circRNAs (positive data) and lncRNAs (negative data). In this study, k-mers, open reading frames (ORFs), and junction sequences covering back-splicing sites were selected as the main features of Pcirc.

#### k-mers

Nucleotides are the most basic elements of gene and transcriptome sequences. The sequences of nucleotides in different genes and transcripts determine different functions, and the corresponding recognition information also includes them, especially the frequency of trinucleotides. Therefore, we extracted the frequency of adjacent nucleotide sequences from the sequence as one of the basic features in this study. For this type of feature, by taking different k-mer values, we first obtained 340 (Σ4k, k = 1, 2, 3, 4) features. The extraction method of each feature f_i_ was as described by formula 1, where fi is the ratio of feature i to the total length of the sequence, xi is the number of times that feature i occurred, k is the k-mer length of feature i, and L is sequence length. Then, we extracted GC content as an extra feature and finally built a vector array with 341 features (formula 2).

1 $$f_{i} = \left( {x_{i} * \, k} \right)/L$$

2 $$X_{{k \text{-}mer}} = \, \left\{ {A\% ,\;T\% ,\;G\% \ldots GAC\% \ldots TTTT\% ,\;GC\;content} \right\}$$

#### ORFs

The open reading frame (ORF) is an important feature in a sequence. Usually, it is the part of a sequence with a protein-coding function. Many studies have shown that the ORF of circRNAs is significantly different from that of coding sequences [27]. Specifically, the ORF-length of the circRNA sequence is shorter, and the ORF-coverage of the total sequence is smaller. Therefore, we use ORF-length and ORF-coverage as a set of features in this study (3). ORF-length refers to the length of the ORF in the sequence, and ORF-coverage refers to the ratio of the length of the ORF to the total length of the sequence.

We first used UGENE (http://ugene.unipro.ru/download.html) to predict the ORF in the sequence and then used a Python script to extract the optimal ORF from the result file and calculate the length ratio of the sequence it occupies. For ORF-coverage, we standardized the value by * 10 when extracting features.

3 $$X_{ORF} = \, \left\{ {ORF\text{-}coverage*10,\;ORF\text{-}length} \right\}$$

#### Splicing junction sequence coding (SJSC)

The process of gene transcription involves many alternative splicing events, and different splicing sites lead to different transcripts, especially in circRNAs. At present, the splice signal GT/AG in circRNAs can be recognized by RBP to form circRNAs. Because the binding regions of RBP are often located upstream and downstream of the back-splicing site, the sequence information upstream and downstream of the back-splicing site may be a useful feature to differentiate circRNAs from other sequences.

**Fig. 2:**
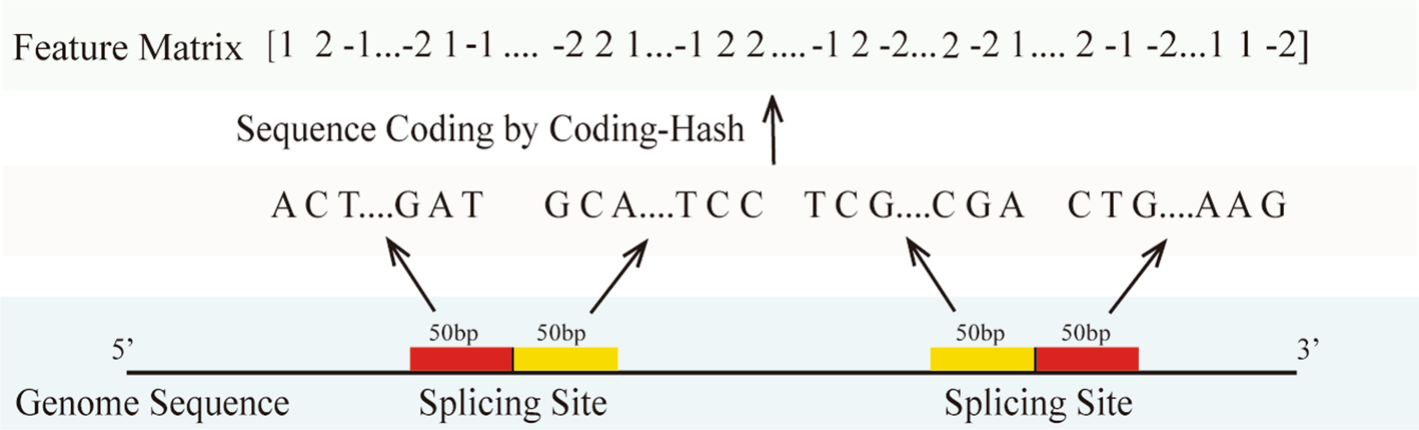
Junction sequence coding with coding-hash {‘A’:1, ‘T’:− 1, ‘C’:2, ‘G’:− 2}

For the back-splicing site, we located two splicing sites in the genome because there are no back-splicing sites for lncRNAs, then extracted the sequences 50 bp upstream and 50 bp downstream of each splicing site in the genome sequence to form a data set, recoded the 100 bp length junction sequence with {‘A’:1, ‘T’:− 1, ‘C’:2, ‘G’:− 2} (Fig. 2), and obtained a one-dimensional array matrix composed of a set of {1, 2, − 1, − 2} that can be recognized by a computer. After all data sets were recoded, a set of corresponding high-dimensional array matrices was generated for training and testing.

### Model measurement

To evaluate our model, we selected some common evaluation parameters: recall rate, precision, accuracy, F1 score, MCC and ROC curve.

4 $$Recall = \frac{{{\text{TP}}}}{{{\text{TP}} + {\text{FN}}}}$$

5 $${\text{Precision }} = \frac{{{\text{TP}}}}{{{\text{TP}} + {\text{FP}}}}$$

6 $$Accuracy = \frac{TP + TN}{{TP + FP + TN + FN}}$$

7 $$F1\text{-}score = \frac{2*Precision*Recall}{{Precision + Recall}}$$

8 $$MCC = \frac{TP*TN - FP*FN}{{\sqrt {\left( {TP + FP} \right)\left( {TP + FN} \right)\left( {TN + FP} \right)\left( {TN + FN} \right)} }}$$

Recall indicates how many positive examples are correctly predicted as positive; precision indicates how many of the predicted positive examples are correct; accuracy indicates the accuracy of the model for all samples; and F1 score indicates the relative stability of the model for positive and negative samples. MCC can evaluate the stability of the model, it will be used in the process of model building and evaluate in the test data. In the formula, TP (true positive) is the number of sequences that are actually circRNAs and correctly predicted as circRNAs; TN (true negative) is the number of sequences that are actually lncRNAs and correctly predicted as lncRNAs; FP (false positive) is the number of sequences that are actually lncRNAs and wrongly predicted as circRNAs; and FN (false negative) is the number of sequences that are actually circRNAs and wrongly predicted as lncRNAs.

## Results

### Algorithm comparison analysis

**Fig. 3:**
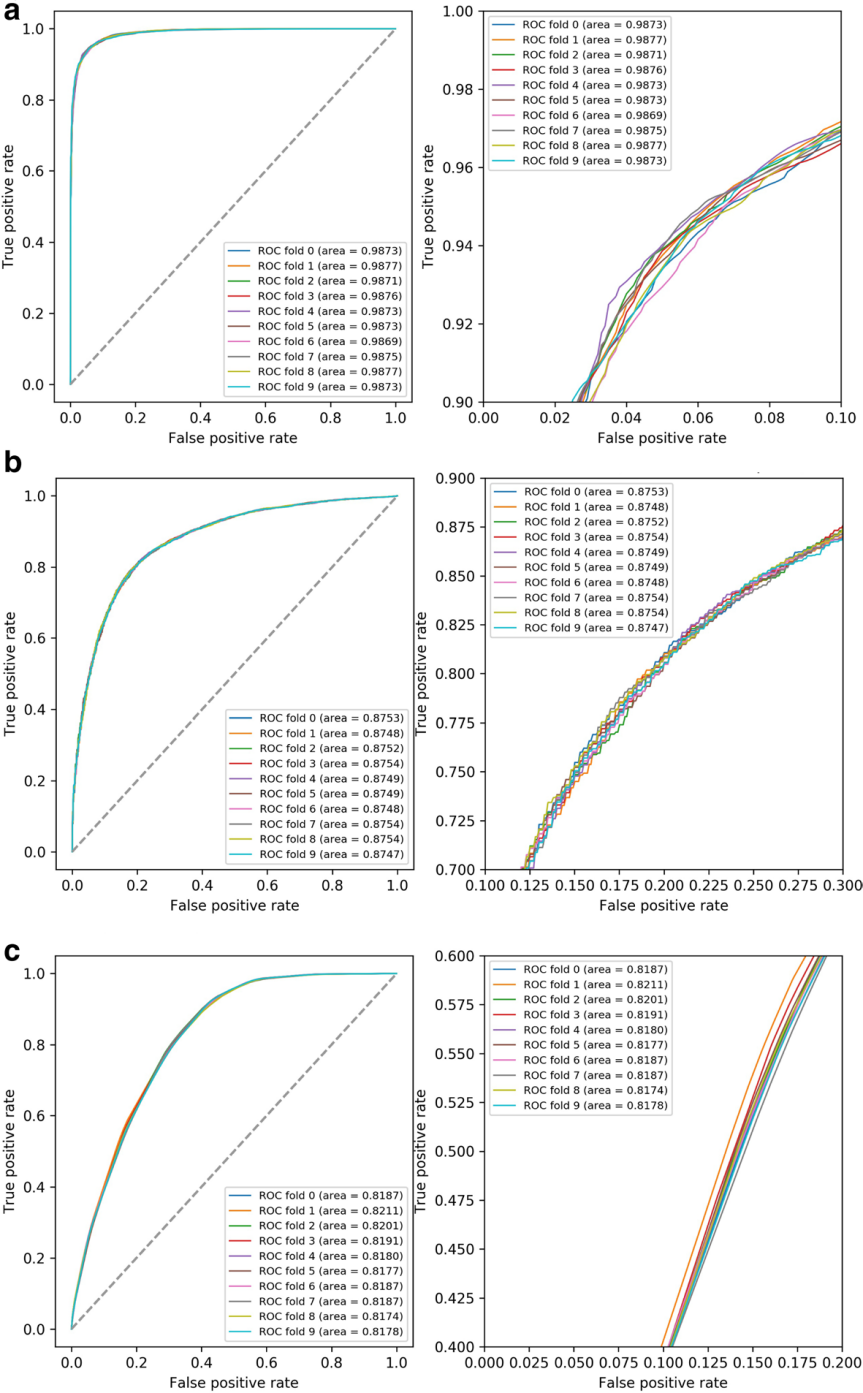
Algorithm evaluation with ROC curve, the right Figure is the ROC curve which zoomed in at loop left of the left figure. **a** The ROC curve of the random forest algorithm. **b** The ROC curve of the Gaussian NB algorithm. **c** The ROC curve of the K-nearest neighbors algorithm

In this study, we chose four popular machine learning algorithms, the K-nearest neighbor algorithm, Gaussian naive Bayes algorithm, support vector machine and random forest algorithm, for comparison. To select the most suitable algorithm, we tested the algorithms on three categories of features. For the modeling test of each type of feature data, we took the tenfold cross-validation, took the average value as the final result, repeated the tenfold cross-validation 10 times, and took the final average value as the final result for comparison (Table 1, Fig. 3). The results showed that the random forest algorithm had the best score, with a minimum score of 0.9433 and the maximum d-values between them less than 0.05, so it was chosen as the best and most stable model building algorithm.

**Table 1:** Algorithm selection

Feature	Algorithm	ACC	PRE	REC	F1-score
k-mers	RF	**0.9584**	0.9448	**0.9738**	**0.9590**
	KNN	0.8465	0.8081	0.9092	0.8556
	GNB	0.8718	0.9279	0.8064	0.8627
	SVM	0.9574	**0.9589**	0.9559	0.9573
ORFs	RF	0.9716	0.9712	0.9721	**0.9716**
	KNN	**0.9717**	**0.9757**	0.9676	0.9716
	GNB	0.9681	0.9603	**0.9767**	0.9684
	SVM	0.9663	0.9611	0.9720	0.9665
SJSC	RF	**0.9433**	**0.9494**	**0.9367**	**0.9429**
	KNN	0.7472	0.7233	0.8013	0.7601
	GNB	0.8036	0.7925	0.8232	0.8074
	SVM	0.8700	0.8553	0.8910	0.8726

For each type of feature, the bold values in this table represent the best score for each evaluation parameter

### Feature combination analysis

**Fig. 4:**
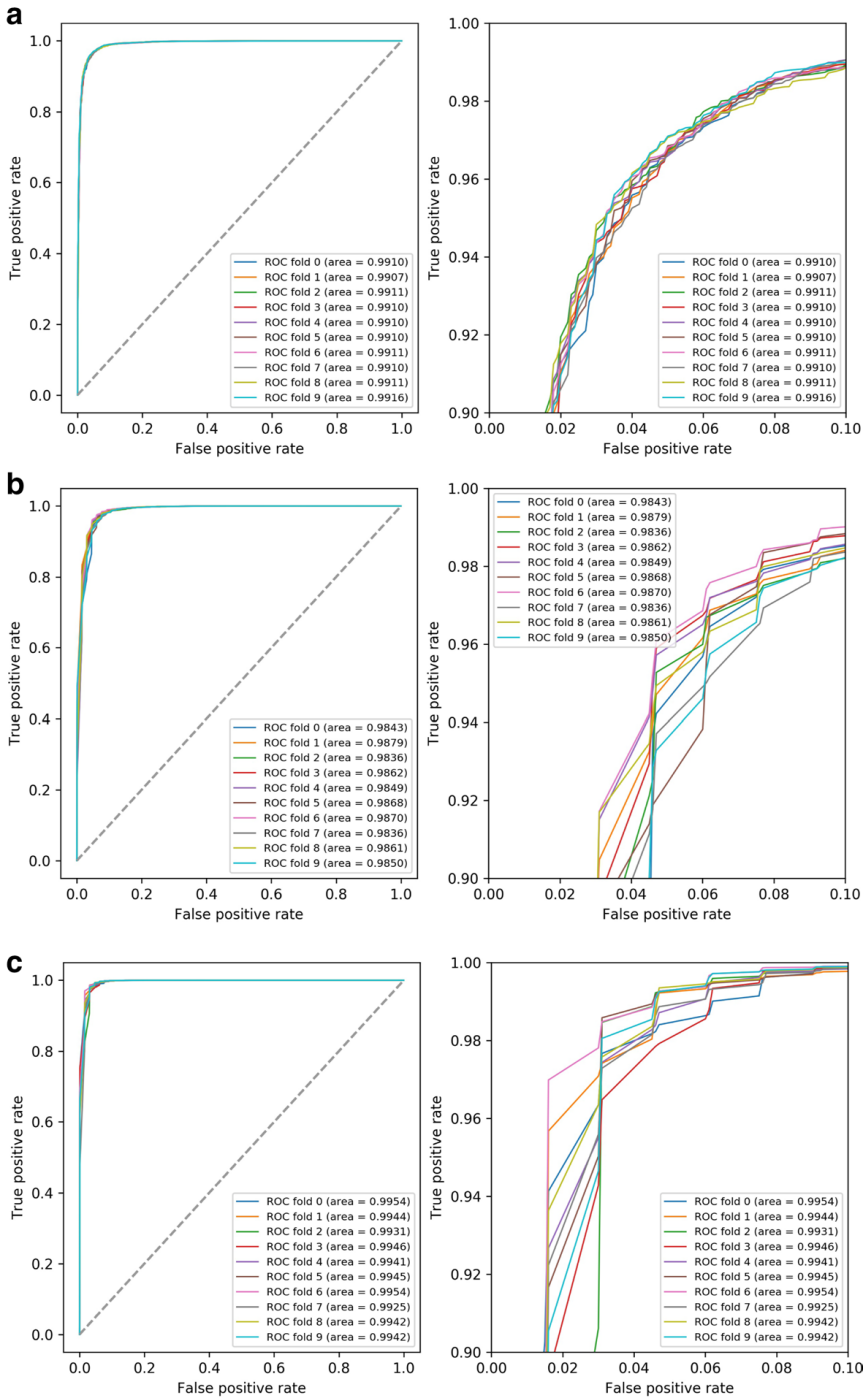
Feature evaluation with ROC curves, the right Figure is the ROC curve which zoomed in at loop left of the left figure. **a** The ROC curves of one feature. **b** The ROC curves of two features combination. **c** The ROC curves of all features

**Fig. 5:**
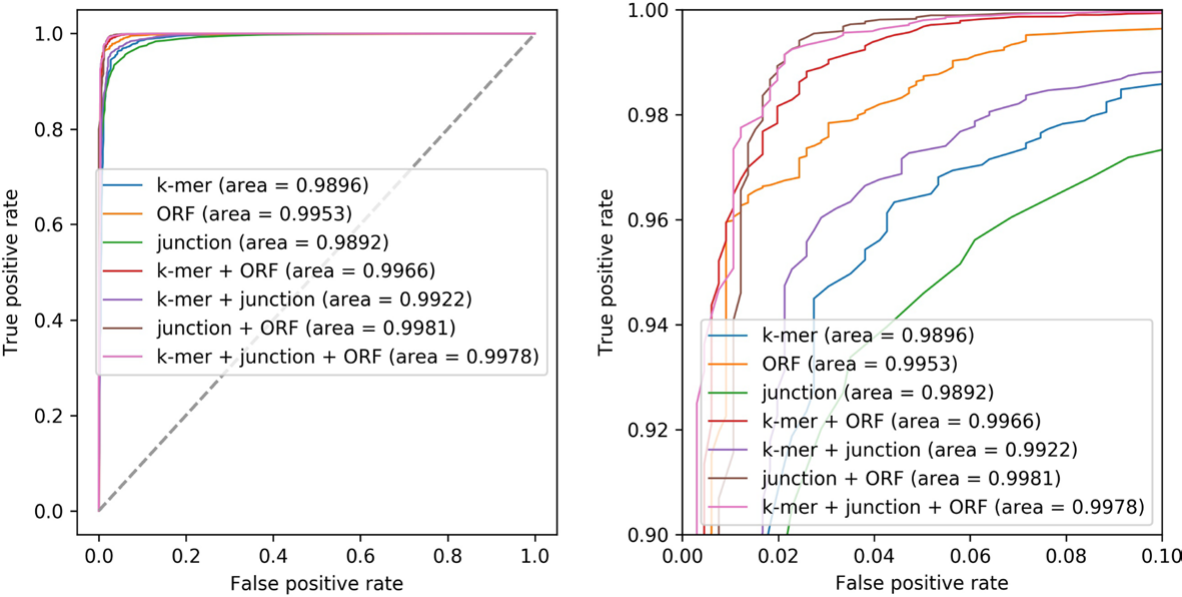
Feature evaluation with ROC curve for test data, the right figure is the ROC curve which zoomed in at loop left of the left figure

In this study, we selected three kinds of features as the main distinguishing features of ML in software to build the model. To test whether the selected features could distinguish the positive and negative categories accurately, we tested the three categories of features separately on the training set (Table 2, Fig. 4). It can be seen that the three categories of features we selected have a good classification effect; the score of every feature was above 0.9, the score of each feature combination was above 0.99, and the parameter evaluation of the combined results was better than that of the single-feature analysis in the ROC curve. The score also increases with the number of features. To avoid overfitting of the model, we also carried out the corresponding test on the test set (Fig. 5). It can be seen that in the test set, the modeling results of the multifeature combination are better and more stable than those of any single type of feature.

**Table 2:** Feature combination test on training data

Feature number	Feature combination	ACC	PRE	REC	F1 score
One	k-mers	0.958400	0.944822	0.973800	0.959044
	ORFs	0.971588	0.971196	0.972075	0.971597
	SJSC	0.943250	0.948866	0.937125	0.942887
	Mean	0.957746	0.954961	0.961000	0.957843
Two	K&O	0.995010	0.995036	0.999887	0.997455
	K&J	0.992597	0.993063	0.999413	0.996227
	J&O	0.995480	0.995401	1.000000	0.997695
	Mean	0.994362	0.994500	0.999767	0.997126
All	K&J&O	0.994818	0.994754	0.999976	0.997358

In this table, K is k-mers, J is SJSC, and O is ORFs

### Model evaluation and application

**Fig. 6:**
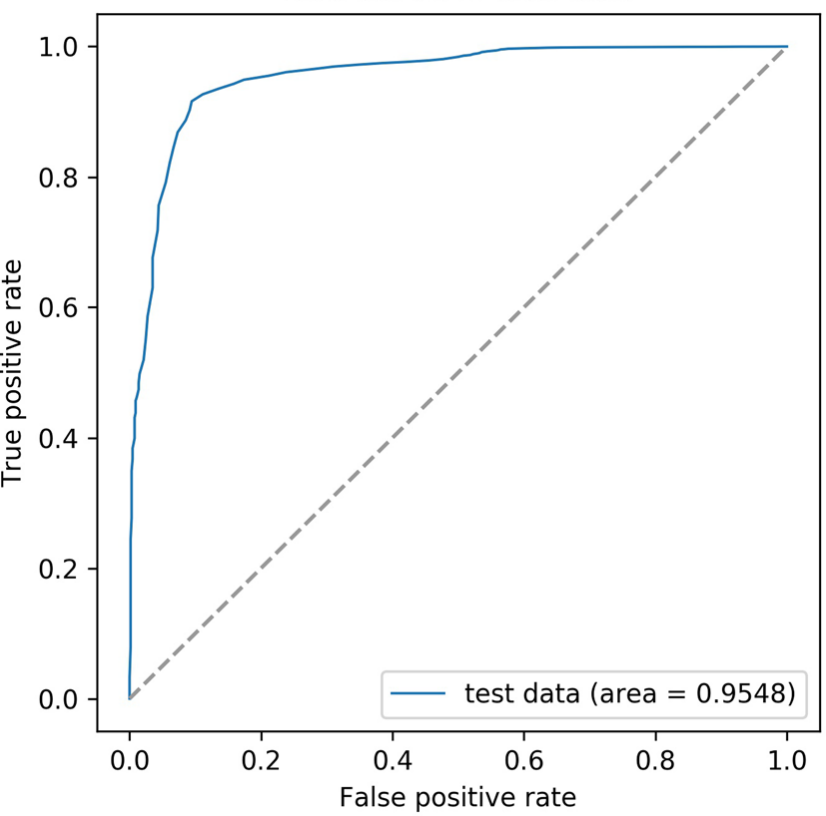
ROC curve of test data

To obtain the best prediction results, we built the model according to the results of the feature combination. To avoid overfitting or underfitting, the fitting effect of the model was evaluated by the method of tenfold cross-validation and precision-recall curve (Additional file 1: Fig. S1, Additional file 2: file 1), and the final model was evaluated on the test set (Table 3, Fig. 6). The results show that our model achieved an accuracy of 0.9936, which shows that our model can classify circRNAs and lncRNAs well.

**Table 3:** The results of the model testing

	ACC	PRE	REC	F1 score	MCC
Model test data	0.9676	0.9904	0.9763	0.9833	0.8749
Ath test data	0.8980	0.9693	0.8220	0.9740	0.8053
Zma test data	0.8130	0.7406	0.9582	0.6644	0.6513

Model test data are the data set from the primary data used to test the ML model

Ath test data are the data set of *Arabidopsis thaliana* used to test the ML model

Zma test data are the data set of *Zea mays* used to test the ML model

Besides only using lncRNA as negative test dataset, we also added the other three types of non-coding RNA including miRNA, snRNA and snoRNA in our negative dataset to test our model. The result showed that the accuracy was 0.8935, and the precision and recall rate reached more than 90% (Additional file 3: file 2.). To test whether our model is also reliable in other plants, we selected circRNA and lncRNA data from the dicotyledon plant Arabidopsis and the monocot plant maize to test our model (Table 3) and achieved accuracy of 0.8980 and 0.8130, respectively. In summary, our results show that our model not only obtains accurate results on the test set for model construction but can also effectively predict circRNAs in other plants.

In order to facilitate plant circRNA researchers and make effective use of the plant circRNA recognition model developed in this study. After model construction, the machine learning model constructed and the programming scripts used in this study were packaged into a locally run circRNA prediction software Pcirc, Pcirc source code and installation instructions are available at https://github.com/Lilab-SNNU/Pcirc, accompanied by its detailed information of code usage.

## Discussion

CircRNAs are a class of circular non-coding RNAs, most of which are larger than 200 nt in length, and lncRNAs are a class of linear noncoding RNAs with length greater than 200nt. For circRNAs and lncRNAs, it is not easily distinguished with only sequence length because the length distribution of circRNA and lncRNAs are almost the same. Combining sequence features with machine learning have been reported to be an effective method for classification long noncoding RNAs [27]. Therefore, starting from the biological characteristics of circRNAs, we constructed a software named Pcirc for predicting plant circRNA by using the machine learning method.

In the process of building machine models, we selected three kinds of features, among which k-mers and ORFs have been widely used in the recognition and prediction of lncRNAs and circRNAs [27, 29]. Because back-splicing sites play an important role in the formation of circRNAs, the upstream and downstream sequences of the back-splicing sites have attracted the attention of scientists. Now researchers began to investigate the formation of circRNAs by perform splicing junction sequence coding (SJSC) [16, 30], but the strategy of SJSC in our method is different from previous method.

In addition to the characteristics associated with splicing sites, we combined the widely used k-mer and ORF features to construct our ML model because they represent the basis of sequences. The k-mer is the basis of sequence diversity. As the value of k increases, it becomes increasingly difficult to find k-mer segments with the same high k values in the same sequence or even in the same genome. At the same time, in a certain range, the larger the k value, the more representative the k-mer fragment will be; however, the additional calculation required by the larger K value is exponentially increased, so we need to better balance the two problems of feature optimization and calculation cost. After several attempts, we chose k_max_ = 4 as the representative value for k-mers.

The order of the four nucleotides contains important biological information, and ORF is a representative type of information. In our study, although this kind of feature is composed of two-dimensional vectors, ORFs also contain rich information after processing. It can be seen from the results of three major algorithm tests and a single-feature type test that this kind of feature has excellent classification ability (Tables 1, 2). Initially, this feature was widely used in the recognition of lncRNAs because the greatest difference between lncRNAs and mRNAs is in coding ability [31]. Currently, circRNAs are mostly regarded as ncRNAs; however, because some circRNAs have been proven to have coding ability, we used this feature for testing, and the results far exceeded our expectations. Probably because of the problem of alternative splicing, the inclusion of more exons in circRNAs than in lncRNAs makes it possible to obtain more ORFs. It is also possible that the circular structure, without clear start and termination sites, is much more translatable than the linear sequence with clear start and termination sites, and thus the ORF feature has very strong classification ability to distinguish circRNAs from lncRNAs. In this study, the ORF feature obtained the best score in the algorithm selection process and the feature combination test (Tables 1, 2). In future research, we will carry out further feature testing, coding analysis and corresponding experimental verification for predicted circRNAs.

## Conclusion

In the context of the lack of tools specifically for plant circRNA prediction, based on rice circRNA and lncRNA data, a machine learning model for plant circRNA recognition was constructed in this study using random forest algorithm, and the model can also be applied to plant circRNA recognition such as Arabidopsis thaliana and maize. At the same time, after the completion of model construction, the machine learning model constructed and the programming scripts used in this study are packaged into a localized circRNA prediction software Pcirc, which is a flexible, lightweight, command-line tool that convenient for plant circRNA researchers to use.

### Availability and requirements

Project name: Pcirc.

Project home page: https://github.com/Lilab-SNNU/Pcirc.

Operating system(s): Unix-based (MacOS, Linux).

Programming language: Python.

Other requirements: Python 3.6.5 or higher, NCBI-blast 2.9.0 or higher, bowtie2 2.2.6 or higher, tophat2 2.1.1 or higher, samtools 0.1.19 or higher, UGENE 1.30.0 or higher, Biopython 1.72, Pandas 0.23.3 or higher, scikit-learn 0.21.2.

License: GGPLv3.

Any restrictions to use by non-academics: License needed.

## Supplementary information

**Additional file 1. Fig. S1**: The precision-recall curve of the model building process.

**Additional file 2. File 1**: The evaluation scores of the model building process.

**Additional file 3. File 2**: The evaluation scores of the test data which include the other three types of non-coding RNA.

**Additional file 4. File 3**: The train set and test set used in the model construction process.

## Data Availability

In this study, all circRNA data, include *Arabidopsis thaliana*, *Oryza sativa*, and *Zea mays*, were downloaded from PlantCircBase (http://ibi.zju.edu.cn/plantcircbase/), release version was v4. All lncRNA data were downloaded from GreeNC (http://greenc.sequentiabiotech.com/wiki/Main_Page), release version was v1.12. The genome data and genome annotation file downloaded from Phytozome (https://phytozome.jgi.doe.gov/pz/portal.html), *Arabidopsis thaliana* genome version was TAIR10, *Oryza sativa* genome version was v7.0, *Zea mays* genome version was AGPv3. The train set and test set used in the model construction process can be obtained from Additional file 4: file 3.

## Abbreviations

ORF: Open reading frame
GNB: Gaussian naive Bayes
TP: True positive
TN: True negative
FP: False positive
FN: False negative
SJSC: Splicing junction sequence coding
KNN: K-nearest neighbors
