# Supplementary figures and images for: PCirc: random forest-based plant circRNA identification software
Source: BMC Bioinformatics. 2021 Jan 6;22:10. doi: 10.1186/s12859-020-03944-1 (PMC7789375; doi:10.1186/s12859-020-03944-1)

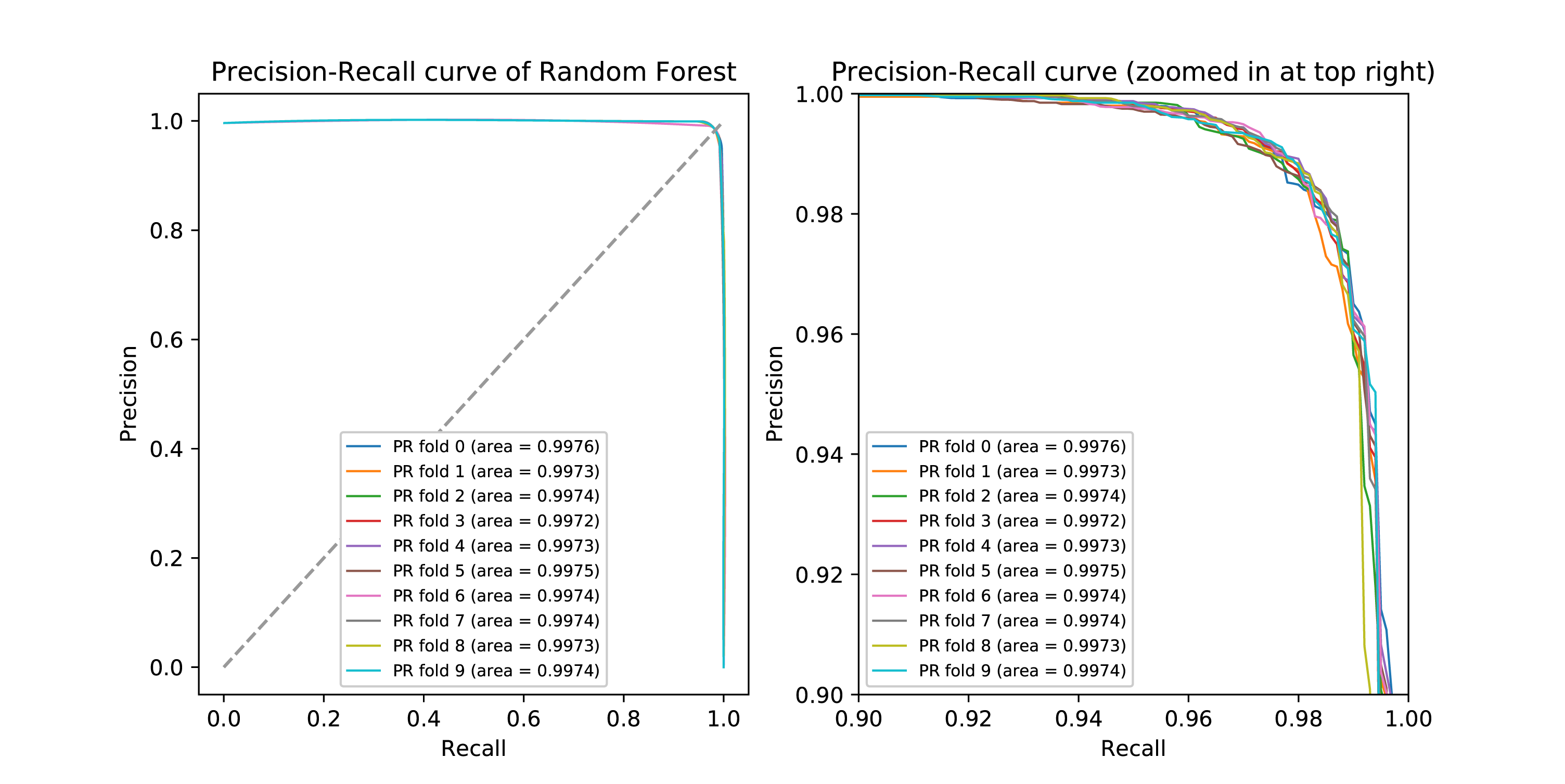

Supplement: Supplementary file 1 — Additional file 1. Fig. S1: The precision-recall curve of the model building process. [file 12859_2020_3944_MOESM1_ESM.tif]
